# PGK1 depletion activates Nrf2 signaling to protect human osteoblasts from dexamethasone

**DOI:** 10.1038/s41419-019-2112-1

**Published:** 2019-11-26

**Authors:** Jinqian Liang, Xiang-yang Zhang, Yun-Fang Zhen, Chong Chen, Haining Tan, Jianhua Hu, Ming-sheng Tan

**Affiliations:** 1 Department of Orthopaedics, Peking Union Medical College Hospital, Beijing, China; 2 Department of Orthopaedics, Tongren Hospital, Shanghai Jiao Tong University School of Medicine, Shanghai, China; 3 The Center of Diagnosis and Treatment for Children’s Bone Diseases, The Children’s Hospital of Soochow University, Suzhou, China; 4 Spinal Surgery, Sino-Japanese Friendship Hospital, Beijing, China

**Keywords:** Proteins, Pathogenesis

## Abstract

Activation of nuclear-factor-E2-related factor 2 (Nrf2) cascade can alleviate dexamethasone (DEX)-induced oxidative injury and death of human osteoblasts. A recent study has shown that phosphoglycerate kinase 1 (PGK1) inhibition/depletion will lead to Kelch-like ECH-associated protein 1 (Keap1) methylglyoxal modification, thereby activating Nrf2 signaling cascade. Here, in OB-6 osteoblastic cells and primary human osteoblasts, PGK1 silencing, by targeted shRNA, induced Nrf2 signaling cascade activation, causing Nrf2 protein stabilization and nuclear translocation, as well as increased expression of ARE-dependent genes (*HO1*, *NQO1*, and *GCLC*). Functional studies demonstrated that PGK1 shRNA largely attenuated DEX-induced oxidative injury and following death of OB-6 cells and primary osteoblasts. Furthermore, PGK1 knockout, by the CRISPR/Cas9 method, similarly induced Nrf2 signaling activation and protected osteoblasts from DEX. Importantly, PGK1 depletion-induced osteoblast cytoprotection against DEX was almost abolished by Nrf2 shRNA. In addition, Keap1 shRNA mimicked and nullified PGK1 shRNA-induced anti-DEX osteoblast cytoprotection. At last we show that PGK1 expression is downregulated in human necrotic femoral head tissues of DEX-taking patients, correlating with HO1 depletion. Collectively, these results show that PGK1 depletion protects human osteoblasts from DEX via activation of Keap1-Nrf2 signaling cascade.

## Introduction

Patients suffering the chronic inflammatory and auto-immune diseases are routinely prescribed with glucocorticoids (dexamethasone/DEX and others^1^). Yet, over-dose and/or sustained DEX administration shall induce profound cytotoxic effects to human osteoblasts, leading to osteoporosis, osteonecrosis, or even non-traumatic bone fractures^2,3^. In vitro studies have demonstrated that to osteoblasts or osteoblastic cells, DEX treatment will exert direct and profound cytotoxicity, leading to subsequent cell apoptosis and necrosis^2,4–7^. By exploring the pathological mechanisms of DEX-induced osteoblast cell death, recent studies have been testing the novel osteoblast-protective strategies^2,4–7^.

DEX treatment in osteoblasts/osteoblastic cells will lead to reactive oxygen species (ROS) production and profound oxidative injury, causing cell death and apoptosis^6,8^. Conversely, inhibition of oxidative stress can protect osteoblasts/osteoblastic cells from DEX^6,8^. The nuclear-factor-E2-related factor 2 (Nrf2) cascade is a vital and endogenous defensive mechanism against oxidative stress^9,10^. Without stimulation, Nrf2 binds to Kelch-like ECH-associated protein 1 (Keap1), subjects to ubiquitination and proteasomal degradation via Cullin 3 (Cul3) E3 ubiquitin ligase^9,10^. Activated Nrf2 protein disassociates with Keap1, being stabilized and accumulated in cytosol, and eventually translocates to cell nuclei, where it binds to ARE (antioxidant response element) loci to promote transcription and expression of key cytoprotective and antioxidant genes^9,10^. These genes, among others, include *heme oxygenase-1* (*HO1*), *NAD(P)H quinone oxidoreductase 1* (*NQO1*), and *γ-glutamyl cysteine ligase catalytic subunit* (*GCLC*, also its modified form, *GCLM*), showing potent antioxidant activity and ability in different human cells^9,10^. Furthermore, agents or stimuli that can modify Keap1’s cysteine residues can also induce Keap1-Nrf2 disassociation and Nrf2 cascade activation^9,11–13^.

Studies have shown that forced activation of Nrf2 cascade shall protect osteoblastic cells/osteoblasts from DEX and other oxidative stresses. For example, Li et al. demonstrated that SC79, a first-in-class Akt activator, protected osteoblasts from DEX via activation of Akt downstream Nrf2 cascade^8^. Liu et al. show that activation of the EGFR-Akt-Nrf2 signaling cascade by icariside II protected osteoblasts from DEX^14^. Compound 991, a novel AMP-activated protein kinase (AMPK) activator, provoked AMPK-dependent Nrf2 signaling to protect osteoblasts from DEX^15^. MIND4-17, by uniquely separating Nrf2-Keap1 complex, attenuated hydrogen peroxide (H_2_O_2_)-induced oxidative stress in osteoblasts^15^. These results show that activation of Nrf2 cascade by pharmacological strategies protects osteoblasts from DEX and other oxidative stress.

Recent literatures have also been using genetic strategies to activate Nrf2 signaling in human osteoblasts. Zhao et al., demonstrated that microRNA-200a (miR-200a) targeted and silenced Keap1, thus activating Nrf2 signaling and protecting osteoblasts from DEX^6^. By targeting tuberous sclerosis complex 1 (TSC1), microRNA-19a (miR-19a) activated mTOR-dependent Nrf2 cascade to alleviate DEX-induced oxidative injury in osteoblasts^7^. In addition, microRNA-455 (miR-445) activated Nrf2 signaling and protected osteoblasts from oxidative stress by targeting Cullin 3, the Nrf2’s E3 ligase^16^. Thus, activation of the Nrf2 cascade, using pharmacological agents or genetic strategies, will exert potent osteoblast cytoprotective actions against DEX-induced oxidative injury.

In the glycolytic pathway, phosphoglycerate kinase 1 (PGK1) is an essential enzyme for ATP generation^17,18^. PGK1 catalyzes the reversible conversion of 1,3-diphosphoglycerate and ADP to 3-phosphoglycerate and ATP^17,18^. A very recent and interesting study has discovered a key role of PGK1 in shutting down Nrf2 cascade activation^19^. PGK1 inhibition or depletion will lead to the accumulation of the reactive metabolite methylglyoxal to modify Keap1^17,19^. The latter will then form a characteristic methylimidazole crosslink between proximal cysteine and arginine residues (MICA), causing Keap1 dimerization, followed by Keap1-Nrf2 disassociation and activation of Nrf2 cascade^17,19^. PKG1 expression and potential functions in human osteoblasts have not been studied thus far. In the present study we will show that PGK1 depletion activates Nrf2 signaling to protect human osteoblasts from DEX.

## Materials and methods

### Chemicals, reagents, and antibodies

DEX, polybrene, puromycin, JC-1 dye, MG-132, cycloheximide (CHX), and 3-[4,5-dimethylthylthiazol-2-yl]-2,5 diphenyltetrazolium bromide (MTT) dye were obtained from Sigma-Aldrich Chemicals (St. Louis, MO). The following antibodies for p44/42 MAPK (Erk1/2, #9102), HO1 (#70081), NQO1 (#3187), Nrf2 (#12721), Keap1 (#8047), α-Tubulin (#2125), and Lamin B1 (#13435) as well as cleaved-poly (ADP-ribose) polymerase (PARP, #5625), cleaved-caspase-3 (#9664) were purchased from Cell Signaling Tech (Shanghai, China). The anti-GCLC antibody (ab55435) and the anti-adenine nucleotide translocase 1 (ANT-1) antibody (ab102032) were provided by Abcam (Shanghai, China). The following antibodies for PGK1 (sc-130335), cyclophilin-D (CyPD, sc-137136), and VDAC1 (sc-390996) were purchased from Santa Cruz Biotech Co. (Santa Cruz, CA). The reagents for cell culturing, including fetal bovine serum (FBS), DMEM, penicillin and streptomycin were obtained from Gibco-BRL Co. (Grand Island, NY). TRIzol and other RNA assay agents, Annexin V, propidium iodide (PI) and cell transfection reagents (Lipofectamine 2000 and others) were from Invitrogen Thermo-Fisher (Shanghai, China). mRNA primers were purchased from Genechem Co. (Shanghai, China). All viral constructs and sequences were also designed and provided by Genechem Co.

### Cell culture

Cultures of the established OB-6^2^ human osteoblastic cells, the primary human osteoblasts (from Dr. Ji at Nanjing Medical University^6^) as well as HEK-293T cells were described early^2,20^. Cells were subjected to mycoplasma and microbial contamination examination every 3 months. Authentication by STR profiling, population doubling time, and morphology were checked as well to confirm the genotype. Primary human osteoblasts were utilized at passages 3–10. The protocols using the primary human cells were approved by the Ethics Committee of all authors institutions, according to the principles of Declaration of Helsinki.

### Quantitative real-time polymerase chain reaction (qPCR)

Following the applied treatment, total RNA was extracted and reverse transcribed as described in ref. ^21^, with qPCR performed by a SYBR Green I real-time PCR kit (Applied Biosystems, Foster City, CA)^22^. The primers for qPCR assays were listed in Table 1. Expression of target mRNA was always normalized to *GAPDH*.

**Table 1:** Sequences of the study.

Genes	Forward primer sequence (5′–3′), qPCR	Reverse primer sequence (5′–3′), qPCR
*PGK1*	CCGCTTTCATGTGGAGGAAGAAG	CTCTGTGAGCAGTGCCAAAAGC
*GCLC*	GGAAGTGGATGTGGACACCAGA	GCTTGTAGTCAGGATGGTTTGCG
*HO1*	CCAGGCAGAGAATGCTGAGTTC	AAGACTGGGCTCTCCTTGTTGC
*NQO1*	AGGCTGGTTTGAGCGAGTTC	ATTGAATTCGGGCGTCTGCTG
*Nrf2*	CACATCCAGTCAGAAACCAGTGG	GGAATGTCTGCGCCAAAAGCTG
*GAPDH*	GTCTCCTCTGACTTCAACAGCG	ACCACCCTGTTGCTGTAGCCAA
*PGK1 sgRNA*	AGCTGGACGTTAAAGGGAAG (Target DNA sequence)	PAM sequence: CGG
*PGK1 shRNA-S1*	AAGAACAACCAGATAACAAACAA	
*PGK1 shRNA-S2*	AAGGATGTTCTGTTCTTGAAGGA	

### Western blotting

The detailed procedures of western blotting were described elsewhere^21,23^. The ImageJ software (NIH, USA) was utilized for the quantification of targeted protein band, with its value normalized to the loading control.

### Mitochondrial isolation and mitochondrial immunoprecipitation (Mito-IP)

The protocol of Mito-IP, examining the mitochondrial cyclophilin-D (CyPD)-adenine nucleotide translocator 1 (ANT-1) association, the indicator of programmed necrosis pathway activation, was described elsewhere^24^.

### shRNA-induced silencing of target genes

A set of two different shRNA oligonucleotides (“S1/S2”, as listed in Table 1) against *human PGK1* were individually annealed and sub-cloned into the GV369 vector (Genechem, Shanghai, China). The construct and the lentivirus packaging constructs (Genechem) were co-transfected to HEK-293T cells, generating PGK1-shRNA-expressing lentivirus. The latter was filtered, enriched and added directly to OB-6 osteoblastic cells or primary human osteoblasts (cultured in 60% cell confluence, in polybrene-containing complete medium). Following selection by puromycin (2.0 μg/mL, for 5–6 passages) stable cells were established, with PGK1 silencing (over 90% knockdown efficiency) verified by qPCR and western blotting. Control cells were transduced with lentiviral scramble control shRNA (“sh-C”). For Nrf2 or Keap1 silencing, the Nrf2 shRNA or the Keap1 shRNA lentiviral particles (Santa Cruz Biotech, Santa Cruz, CA) were individually added to cultured OB-6 cells. After puromycin selection the stable cells were established, with Nrf2 or Keap1 silencing verified by qPCR and western blotting assays.

### PGK1 knockout (KO)

The targeted small guide RNA (sgRNA, as listed in Table 1) against *human PGK1* was annealed into a CRISPR/Cas9 PX458-GFP construct (a gift from Dr. Hu^25^). The construct was tranduced to OB-6 osteoblastic cells (cultured at 60% cell confluence) by using the Lipofectamine 2000 protocol. The transfected cells were further subjected to fluorescence-activated cell sorting (FACS) GFP sorting, with single cells distributed to the 24-well plates for 2 more weeks. Stable cells were subjected to PGK1 expression screen. PGK1 KO was verified by qPCR and western blotting assays. Control cells were transduced with the CRISPR/Cas9 PX458-GFP construct with scramble non-sense sgRNA (“sg-C”).

### MTT viability analyses

At a density of 3 × 10^3^ cells per well OB-6 cells or the primary osteoblasts were seeded into 96-well tissue-culture plates. After the applied DEX treatment, cell viability was tested by the MTT dye assay. MTT optical density (OD) was measured at the test-wavelength of 490 nm.

### Lactate dehydrogenase (LDH) release assay

OB-6 cells or primary osteoblasts were seeded into 12-well tissue-culture plates (at a density of 5 × 10^4^ cells in each well). Following the applied DEX treatment a two-step simple LDH assay kit (Takara, Tokyo, Japan) was utilized to quantify LDH contents in the medium, always normalized to total LDH levels.

### JC-1 assaying of mitochondrial depolarization

With mitochondrial depolarization JC-1 aggregating in mitochondria forms green monomers^26^. OB-6 osteoblastic cells or primary human osteoblasts were seeded into 12-well tissue-culture plates (at a density of 5 × 10^4^ cells in each well). Following the applied DEX treatment OB-6 cells or the primary osteoblasts were stained with JC-1 (5 μg/mL, 12 min under the dark) and tested immediately by a fluorescence spectrofluorometer at 550 nm. The representative JC-1 images, merging the green fluorescence image (at 550 nm) together with the red fluorescence image (at 650 nm), were presented as well.

### Superoxide detection

OB-6 osteoblastic cells or primary human osteoblasts were seeded into six-well tissue-culture plates (at 1 × 10^5^ cells in each well). Following the indicated DEX treatment, a superoxide colorimetric assay kit (BioVision, San Francisco, CA) was applied to measure cellular superoxide contents. In brief, the superoxide detection reagent (100 µL/well) was added to cultured cells for 30 min, with the superoxide absorbance measured at 450 nm.

### Glutathione content assay

OB-6 cells or primary human osteoblasts were seeded into six-well tissue-culture plates (at 1 × 10^5^ cells in each well). We compared the ratio of reduced glutathione (GSH) with oxidized disulfide form glutathione (GSSG)^27^, using a previously-described protocol^27^.

### Annexin V assay

OB-6 cells or primary osteoblasts were seeded into six-well plates (at a density of 3 × 10^5^ cells per well). Following the applied DEX treatment cell apoptosis was tested by Annexin V-PI assay, using the described protocol^28^.

### Histone-DNA ELISA assay

OB-6 cells were seeded into 96-well plates at a density of 3 × 10^3^ cells per well. Following the applied DEX treatment the apoptosis intensity was quantified using a histone-DNA ELIA kit (Roche, Palo Alto, CA)^29^, with ELISA absorbance tested at 450 nm.

### Human tissue collection and analyses

As described in refs. ^4,21^, from twelve (12) written-informed consent DEX-treated patients with femoral head resection, the necrotic femoral head tissues and surrounding normal femoral head tissues were collected, dissolved in the tissue lysis buffer, and tested by qPCR and western blotting. The clinical investigations were conducted according to the principles of Declaration of Helsinki. The protocols of this study were approved by Ethics Committee of Peking Union Medical College Hospital.

### Statistical analysis

The investigator was blinded to the group allocation during the experiments. Data were presented as means ± standard deviation (SD). Statistical analyses were performed in data with normal distribution by one-way analysis of variance (ANOVA) followed by multiple comparisons with Bonferroni’s post hoc test (SPSS 18.0; SPSS Co., Chicago, IL). The two-tailed unpaired T test (Excel 2007) was utilized when comparing two specific treatment groups. *p* values < 0.05 were considered statistically significant^28^.

## Results

### PGK1 silencing activates Nrf2 signaling in human osteoblasts

**Fig. 1:**
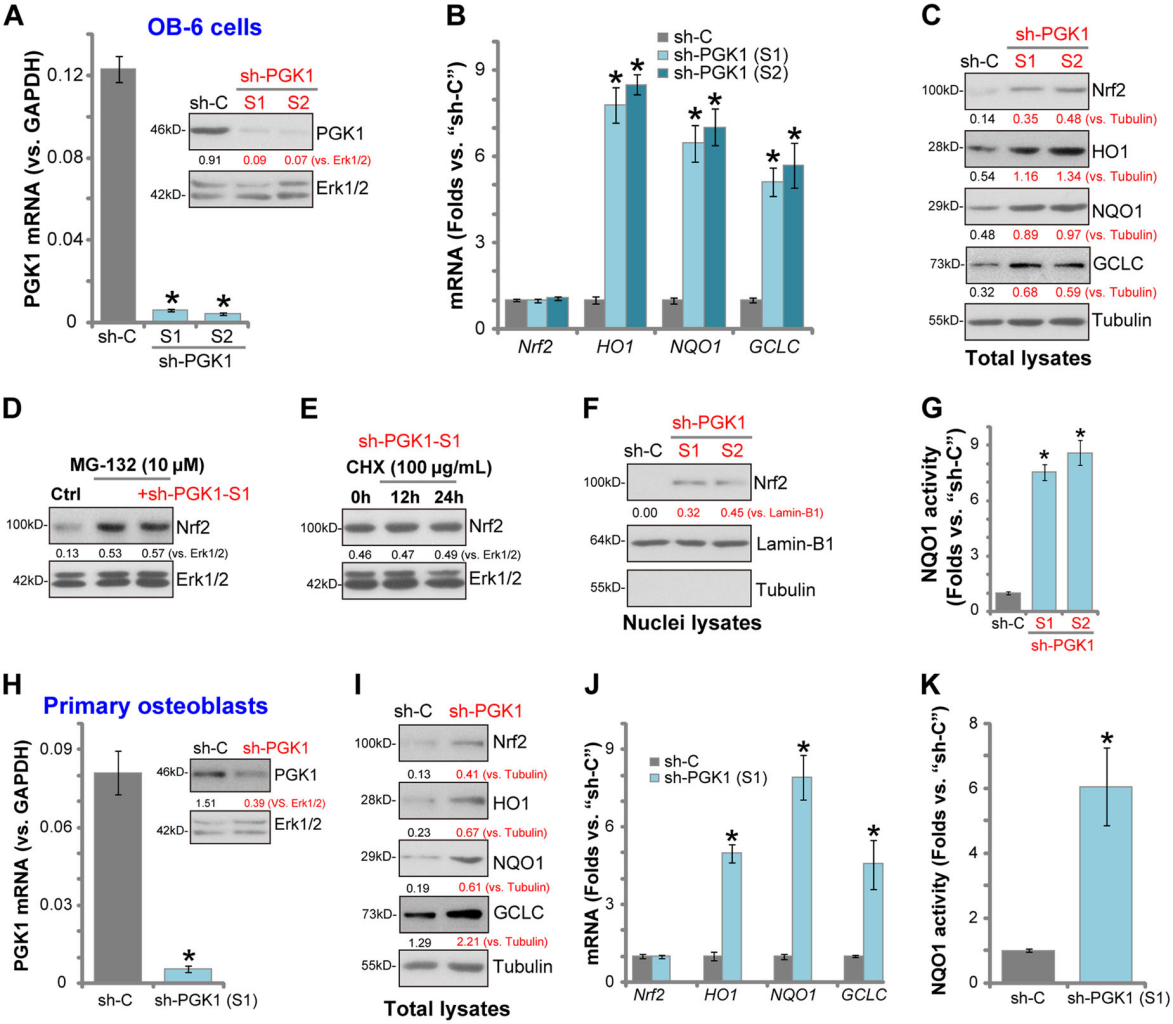
PGK1 silencing activates Nrf2 signaling in human osteoblasts. Expression of listed genes (mRNAs and proteins) in stable OB-6 osteoblastic cells, with applied PGK1 shRNA (“sh-PGK1-S1/S2”) or the non-sense control shRNA (“sh-C”), were tested by qPCR and western blotting assays (**a**–**c**, **f**). The relative NQO1 activity was tested as well (**g**). OB-6 cells were treated with MG-132 (10 μM) or plus sh-PGK1-S1 infection (“+sh-PGK1-S1”), after 24 h Nrf2 and Erk1/2 (the loading control) protein expression in total cell lysates was shown (**d**). Stable OB-6 cells with sh-PGK1-S1 were treated with cycloheximide (CHX, 100 μg/mL) for 12 and 24 h, Nrf2 and Erk1/2 protein expression in total cell lysates was shown (**e**). The primary human osteoblasts were transduced with lentiviral PGK1 shRNA (“sh-PGK1-S1”) or the lentiviral non-sense control shRNA (“sh-C”), expression of listed genes (**h**–**j**) and the relative NQO1 activity (**k**) were tested. Expression of listed proteins was quantified, normalized to the loading control (**a**, **c**–**e**, **h**, **i**). Data were expressed as mean ± standard deviation (SD, *n* = 5). *p ***<*** 0.05 vs. “sh-C” cells. Experiments were repeated four times, with similar results obtained.

First we tested whether PGK1 silencing could induce Nrf2 cascade activation in human osteoblasts. The differentiated OB-6 human osteoblastic cells^30,31^ were transduced with the lentiviral PGK1 shRNA (“sh-PGK1-S1/S2”, with non-overlapping sequence), and selected by puromycin to establish the stable cell lines. Analyzing mRNA expression, by qPCR, show that *PGK1 mRNA* levels decreased over 95% in sh-PGK1-expressing stable cells (*p* < 0.05 vs. cells with non-sense control shRNA/“sh-C”) (Fig. 1a). PGK1 protein levels were downregulated as well by the applied PGK1 shRNA (Fig. 1a). To examine Nrf2 signaling we show that mRNA expression of Nrf2-dependent genes, including *HO1*, *NQO1*, and *GCLC*, was significantly increased in PGK1-silenced OB-6 cells (*p* < 0.05 vs. cells with “sh-C”, Fig. 1b). Their protein levels were also increased (Fig. 1c). *Nrf2 mRNA* level was not significantly affected by PGK1 silencing in OB-6 cells (Fig. 1b), whereas its protein level was significantly increased (Fig. 1c), indicating Nrf2 protein stabilization and accumulation.

Significantly, PGK1 silencing by sh-PGK1-S1 failed to further increase Nrf2 protein expression in OB-6 cells with pre-treatment of MG-132, a well-known proteasome inhibitor (Fig. 1d). In sh-PGK1-S1-expressing OB-6 cells treatment with cycloheximide (CHX), the protein synthesis inhibitor, had no significant effect on Nrf2 protein expression (Fig. 1e). These results indicate that PGK1 shRNA-induced Nrf2 protein upregulation is due to protein stabilization. In PGK1-silenced OB-6 cells, the stabilized Nrf2 translocated to the nuclei, evidenced by increased Nrf2 protein in cell nuclei lysates (Fig. 1f). The NQO1 activity was also significantly increased by PGK1 shRNA (Fig. 1g). These results together show that PGK1 silencing induced Nrf2 protein stabilization, nuclear translocation and activation in OB-6 cells.

In the primary human osteoblasts the applied lentiviral PGK1 shRNA (sh-PGK1-S1) potently downregulated *PGK1 mRNA* and protein expression (Fig. 1h). Further studies show that PGK1 silencing induced Nrf2 protein stabilization (Fig. 1i), mRNA and protein expression of Nrf2-dependent genes (*HO1*, *NQO1*, and *GCLC*, Fig. 1i, j) as well as an increase of NQO1 activity (Fig. 1k). Therefore, PGK1 silencing induced Nrf2 cascade activation in the primary human osteoblasts.

### PGK1 silencing protects human osteoblasts from DEX-induced death and apoptosis

Previous studies have shown that DEX induces ROS production and significant oxidative injury in human osteoblasts, leading to following cell death and apoptosis^6,8,14,15^. On the contrary, activation of Nrf2 cascade can protect osteoblasts from DEX-induced oxidative stress^6,8,14,15^. We have shown that PGK1 silencing by targeted shRNA-induced Nrf2 cascade activation, we next tested whether it could protect osteoblasts from DEX.

**Fig. 2:**
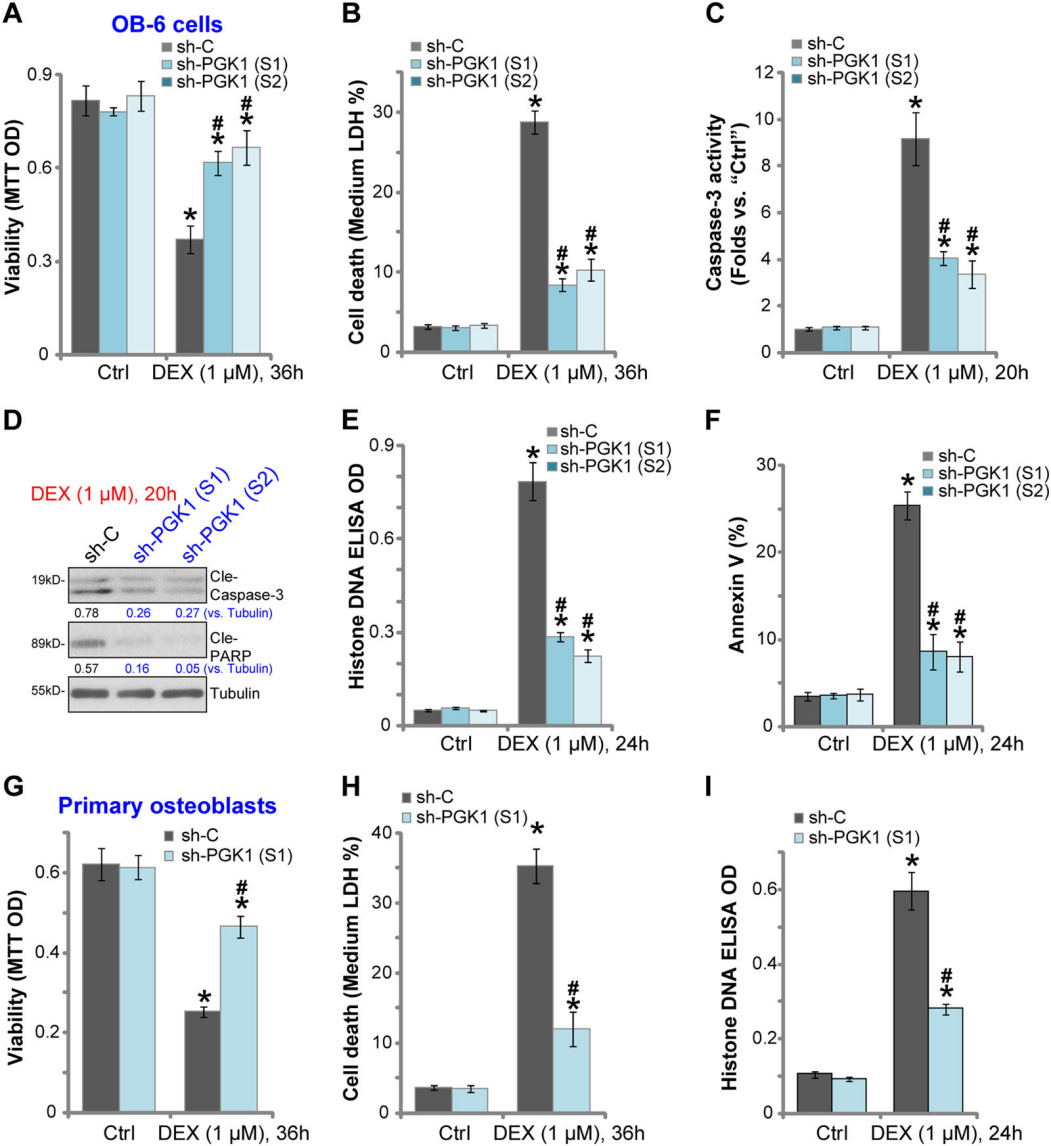
PGK1 silencing protects human osteoblasts from DEX-induced death and apoptosis. Stable OB-6 osteoblastic cells (**a**–**f**) or primary human osteoblasts (**g**–**i**) with applied PGK1 shRNA (“sh-PGK1-S1/S2”) or the non-sense control shRNA (“sh-C”) were treated with or without DEX (1 μM) for applied time periods, cell viability (MTT assay; **a** and **g**) and cell death (LDH medium release; **b** and **h**) were tested; Cell apoptosis activation was tested by the listed assays (**c**–**f**, **i**). Expression of listed proteins was quantified, normalized to the loading control (**d**). Data were expressed as mean ± standard deviation (SD, *n* = 5). “Ctrl” represents untreated control group (Same for all Figures). *p < 0.05 vs. “sh-C” cells with “Ctrl” treatment. ^#^*p* < 0.05 vs. “sh-C” cells with DEX treatment. Experiments in this figure were repeated four times, and similar results were obtained.

In line with the previous findings^21,23,32,33^, in “sh-C” control OB-6 cells DEX treatment induced significant viability (MTT OD) reduction (Fig. 2a), cell death (medium LDH release, Fig. 2b), caspase-3-poly (ADP)-ribose polymerase (PARP) activation (Fig. 2c, d). Furthermore, apoptosis activation in DEX-treated control OB-6 cells was evidenced by increased Histone-bound DNA accumulation (Fig. 2e) and Annexin V staining (Fig. 2f). Significantly, DEX-induced cytotoxicity and apoptosis were significantly attenuated in stable OB-6 cells-expressing PGK1 shRNA (Fig. 2a–f). Therefore, PGK1 silencing protected OB-6 cells from DEX.

The primary human osteoblasts with PGK1 shRNA were also protected from DEX, showing significantly decreased viability reduction (Fig. 2g), cell death (LDH medium release, Fig. 2h) and apoptosis (Histone-bound DNA accumulation, Fig. 2i), when compared with the control osteoblasts (with “sh-C”) with same DEX treatment (Fig. 2g–i). Thus PGK1 silencing protected primary human osteoblasts from DEX-induced death and apoptosis.

### PGK1 silencing attenuates DEX-induced oxidative stress and programmed necrosis in human osteoblasts

**Fig. 3:**
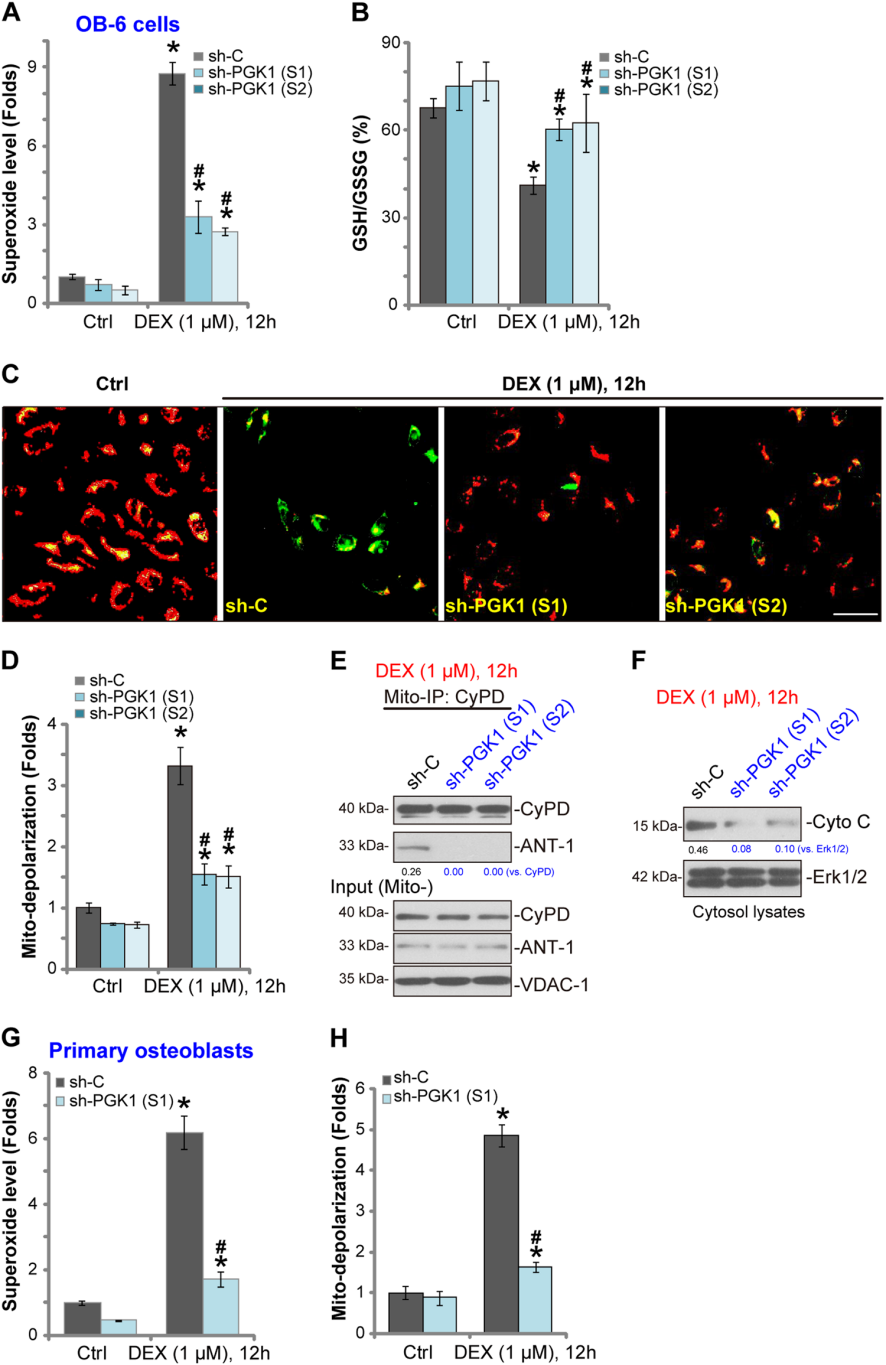
PGK1 silencing attenuates DEX-induced oxidative stress and programmed necrosis in human osteoblasts. Stable OB-6 human osteoblastic cells (**a**–**f**) or primary human osteoblasts (**g**, **h**) with applied PGK1 shRNA (“sh-PGK1-S1/S2”) or the non-sense control shRNA (“sh-C”), were treated with or without DEX (1 μM) for applied time periods, oxidative stress (**a**, **b**, **g**) and programmed necrosis (**c**–**f**, **h**) were tested by the listed assays mentioned in the text. For mitochondrial depolarization assay, JC-1 green intensity was examined at 550 nm via a fluorescence spectrofluorometer (results normalized to the control level), and the representative JC-1 fluorescence images, merging both green and red fluorescence pictures, were presented (**c**, **d**). Expression of listed proteins was quantified, normalized to the loading control (**e**, **f**). Data were expressed as mean ± standard deviation (SD, *n* = 5). *p < 0.05 vs. “sh-C” cells with “Ctrl” treatment. ^#^*p* < 0.05 vs. “sh-C” cells with DEX treatment. Experiments in this figure were repeated four times, and similar results were obtained. Bar = 100 μm (**c**).

The potential role of PGK1 shRNA on DEX-induced oxidative stress was examined next. As demonstrated, in “sh-C” control OB-6 cells DEX treatment induced significant superoxide accumulation (Fig. 3a) and GSH/GSSG ratio reduction (Fig. 3b), indicating a significant oxidative injury. Such actions by DEX were largely attenuated by the applied PGK1 shRNA (Fig. 3b)

Besides apoptosis, DEX-provoked ROS production concurrently induces mitochondrial programmed necrosis pathway in human osteoblasts, as reported by other studies^8,24,30,34,35^. Here we show that DEX (1 μM) treatment in control OB-6 cells induced significant mitochondrial depolarization (JC-1 green fluorescence accumulation^26,36^, Fig. 3c, d), mitochondrial CyPD-ANT-1 association (an initial step of programmed necrosis progression^8,24,35^, Fig. 3e), and cytosol cytochrome C release (Fig. 3f), showing activation of the programmed necrosis pathway^8,24,35^. Importantly, PGK1 silencing by targeted shRNA largely attenuated DEX-induced programmed necrosis activation (Fig. 3c–f). In the primary human osteoblasts DEX-induced superoxide accmulation (Fig. 3g) and mitochondrial depolarization (Fig. 3h) were largely inhibited by PGK1 shRNA as well. Collectively these results show that PGK1 shRNA inhibited DEX-induced oxidative stress and programmed necrosis in human osteoblasts.

### PGK1 knockout activates Nrf2 signaling and protects osteoblasts from DEX

**Fig. 4:**
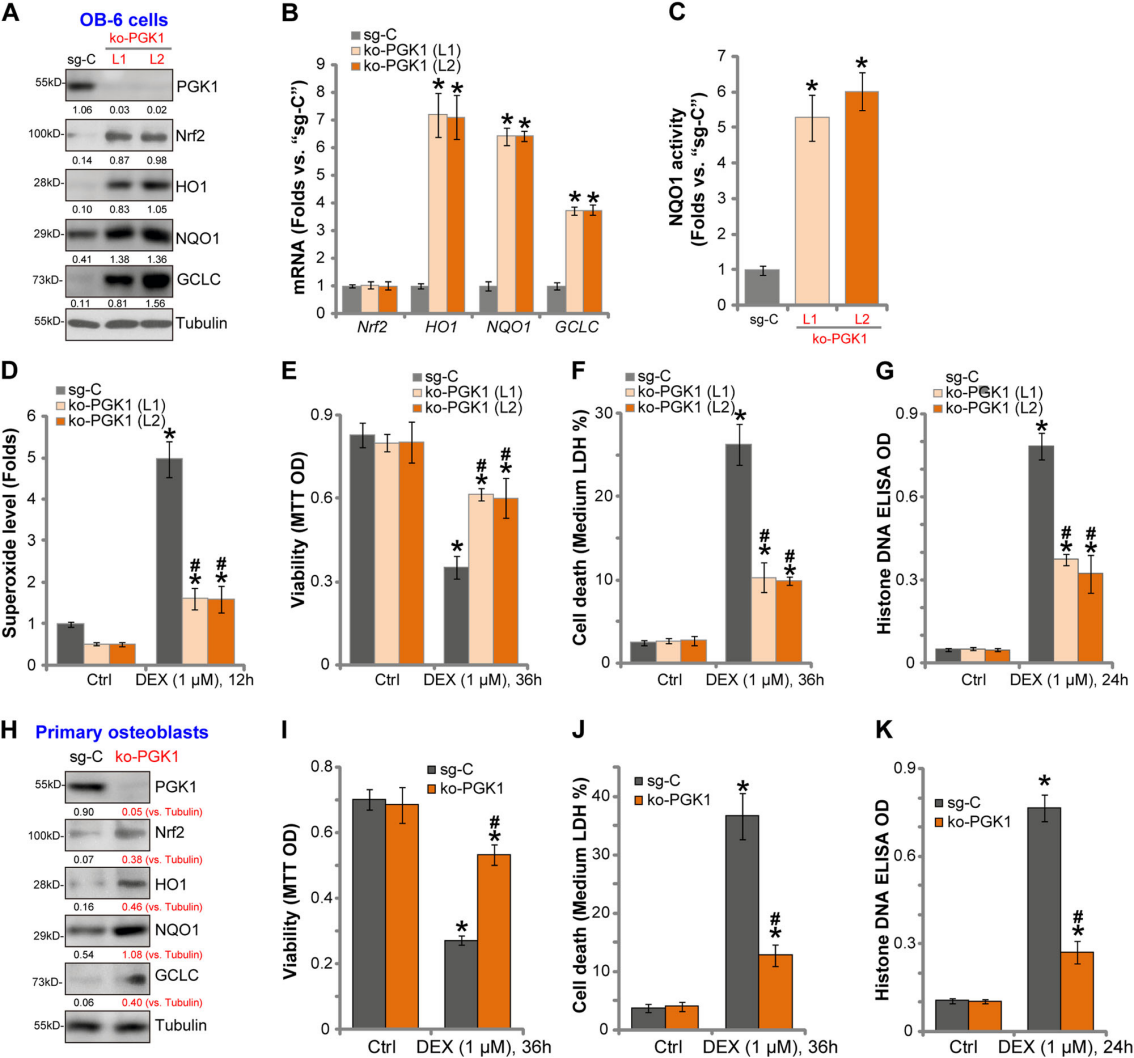
PGK1 knockout activates Nrf2 signaling and protects osteoblasts from DEX. Stable OB-6 osteoblastic cells (**a**–**g**) or primary human osteoblasts (**h**–**k**) with the CRISPR/Cas9-PGK1 knockout construct (“ko-PGK1”) or the CRISPR/Cas9 sgRNA control construct (“sg-C”) were established, expression of listed genes (**a**, **b**, **h**) was shown. The relative NQO1 activity was also tested (**c**). Cells were further treated with or without DEX (1 μM) for applied time periods, cellular superoxide levels were shown (**d**); cell viability (**e**, **i**), death (**f**, **j**), and apoptosis (**g**, **k**) were examined by MTT, LDH release, and Histone-DNA ELISA assays, respectively. Expression of listed proteins was quantified, normalized to the loading control (**a**, **h**). Data were expressed as mean ± standard deviation (SD, *n* = 5). *p < 0.05 vs. “sg-C” cells with “Ctrl” treatment. ^#^*p* < 0.05 vs. “sg-C” cells with DEX treatment. Experiments in this figure were repeated three times, and similar results were obtained.

To further support our hypothesis a CRISPR/Cas9-PGK1 knockout construct was transduced to OB-6 cells. Subject to selection with puromycin two stable OB-6 cell lines with the construct were established, ko-PGK1-L1 and ko-PGK1-L2. In these stable cells PGK1 protein expression was completely depleted (Fig. 4a), leading to Nrf2 protein stabilization, as well as mRNA and protein expression of Nrf2-dependent genes (*HO1*, *NQO1*, and *GCLC*) (Fig. 4a, b). As compared the control cells (with sgRNA control or sg-C), the NQO1 activity was significantly increased in ko-PGK1 OB-6 cells (Fig. 4c). Functional studies demonstrated that DEX-induced oxidative stress, reflected by superoxide accumulation, was largely inhibited in ko-PGK1 OB-6 cells (Fig. 4d). Furthermore, DEX-induced viability (MTT OD) reduction (Fig. 4e), cell death (LDH medium release, Fig. 4f) and apoptosis (Histone-bound DNA accumulation, Fig. 4g) were largely attenuated by PGK1 KO. These results show that CRISPR/Cas9 induced PGK1 KO activated Nrf2 signaling and inhibited DEX-induced oxidative injury in OB-6 osteoblastic cells.

In the primary human osteoblasts, the applied CRISPR/Cas9-PGK1 KO construct led to PGK1 depletion (Fig. 4h), Nrf2 protein stabilization (Fig. 4h) as well as protein expression of HO1, NQO1, and GCLC (Fig. 4h). Significantly DEX-induced viability reduction (Fig. 4i), cell death (Fig. 4j), and apoptosis (Fig. 4k) were largely alleviated in PGK1 KO human osteoblasts. Collectively, these results show that PGK1 KO activated Nrf2 signaling and protected human osteoblasts from DEX.

### PGK1 depletion-induced osteoblast cytoprotection against DEX is through activation of Keap1-Nrf2 cascade

**Fig. 5:**
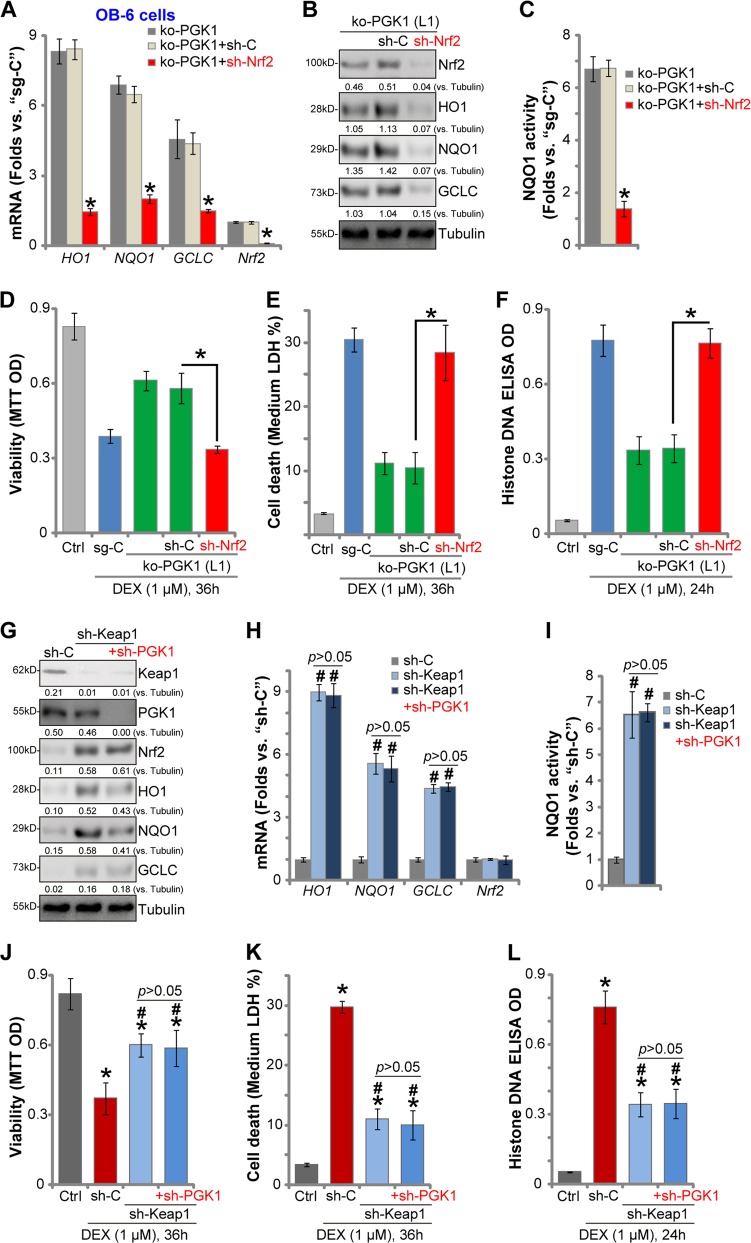
PGK1 depletion-induced osteoblast cytoprotection against DEX is through activation of Keap1-Nrf2 cascade. OB-6 cells with the CRISPR/Cas9-PGK1-knockout construct (“ko-PGK1”) were further transduced with Nrf2 shRNA (“+sh-Nrf2”) or the non-sense control shRNA (“+sh-C”), stable cells were established via selection by puromycin, relative expression of Nrf2 pathway genes was shown (**a**, **b**). The NQO1 activity was also tested (**c**). Above cells and the control cells (with CRISPR/Cas9 sgRNA control construct/sg-C) were further treated with or without DEX (1 μM) for applied time periods, cell viability (**d**), death (**e**) and apoptosis (**f**) were examined by MTT, LDH release, and Histone-DNA ELISA assays, respectively. Expression of the listed genes in the stable OB-6 cells with the non-sense control shRNA (“sh-C”), the lentiviral Keap1 shRNA (“sh-Keap1”), or together with the PGK1 shRNA (“sh-PGK1-S1”, sh-Keap1 + sh-PGK1) was shown (**g**, **h**), the relative NQO1 activity was tested as well (**i**). The above cells were treated with or without DEX (1 μM) for applied time periods, cell viability (**j**), death (**k**), and apoptosis (**l**) were tested similarly. Expression of listed proteins was quantified, normalized to the loading control (**b**, **g**). Data were expressed as mean ± standard deviation (SD, *n* = 5). *p < 0.05 vs. “ko-PGK1” cells (**a**, **c**); *p < 0.05 (**d**–**f**); ^#^*p* < 0.05 vs. “sh-C” cells (**h**–**l**). *p < 0.05 vs. “Ctrl” treatment (**j**–**l**). *p* > 0.05 stands for no statistical difference. Experiments in this figure were repeated three times, and similar results were obtained.

We have shown that PGK1 depletion induced Nrf2 signaling cascade activation and protected osteoblasts from DEX. To test the link between the two, in PGK1 knockout OB-6 cells [ko-PGK1(-L1), see Fig. 4] the lentiviral shRNA was utilized to stably knockdown Nrf2 (“+sh-Nrf2”). As shown PGK1 KO-induced mRNA (Fig. 5a) and protein (Fig. 5b) expression of *HO1*, *NQO1*, and *GCLC* was almost completely blocked by Nrf2 shRNA in OB-6 cells. Furthermore, the Nrf2 protein stabilization in ko-PGK1 cells was reversed by the Nrf2 shRNA (Fig. 5b). The increase of NQO1 activity in ko-PGK1 cells was also largely attenuated by Nrf2 shRNA (Fig. 5c). In OB-6 cells PGK1 KO significantly inhibited DEX-induced viability reduction (MTT OD decrease, Fig. 5d), cell death (LDH medium release, Fig. 5e) and apoptosis (Histone-DNA accumulation, Fig. 5f). Such actions were also reversed by Nrf2 shRNA (Fig. 5d–f). These results suggest that Nrf2 activation is required for PGK1 KO-induced osteoblast cytoprotection against DEX.

PGK1 depletion-induced Nrf2 cascade activation requires Keap1 methylglyoxal modification and dimerization^17,19^, we therefore hypothesized that Keap1 silencing should mimic PGK1 depletion-induced actions. OB-6 osteoblastic cells were transduced with the the lentiviral Keap1 shRNA, resulting in Keap1 depletion (Fig. 5g). In Keap1-silenced cells *HO1*, *NQO1*, and *GCLC* mRNA (Fig. 5h) and protein (Fig. 5g) expression and the NQO1 activity (Fig. 5i) were significantly increased (vs. “sh-C” control cells), mimicking PGK1 depletion-induced actions. As compared with control OB-6 cells, Keap1-shRNA-expressing cells were protected from DEX, showing significantly reduced viability reduction (Fig. 5j), cell death (Fig. 5k) and apoptosis (Fig. 5l). Importantly, in Keap1-silenced OB-6 cells PGK1 silencing by sh-PGK1-S1 (Fig. 5g) failed to further increase Nrf2 signaling activation (Fig. 5g–i), nor it did offer further cytoprotection against DEX (Fig. 5j–l). Notably, Keap1 silencing did not affect PGK1 expression in OB-6 cells (Fig. 5g). Therefore, Keap1 silencing mimicked and nullified PGK1 shRNA-induced osteoblast cytoprotection against DEX, suggesting that PGK1 depletion-induced osteoblast cytoprotection against DEX is through activation of Keap1-Nrf2 cascade.

### PGK1 downregulation in human necrotic femoral head tissues correlates with HO1 depletion

**Fig. 6:**
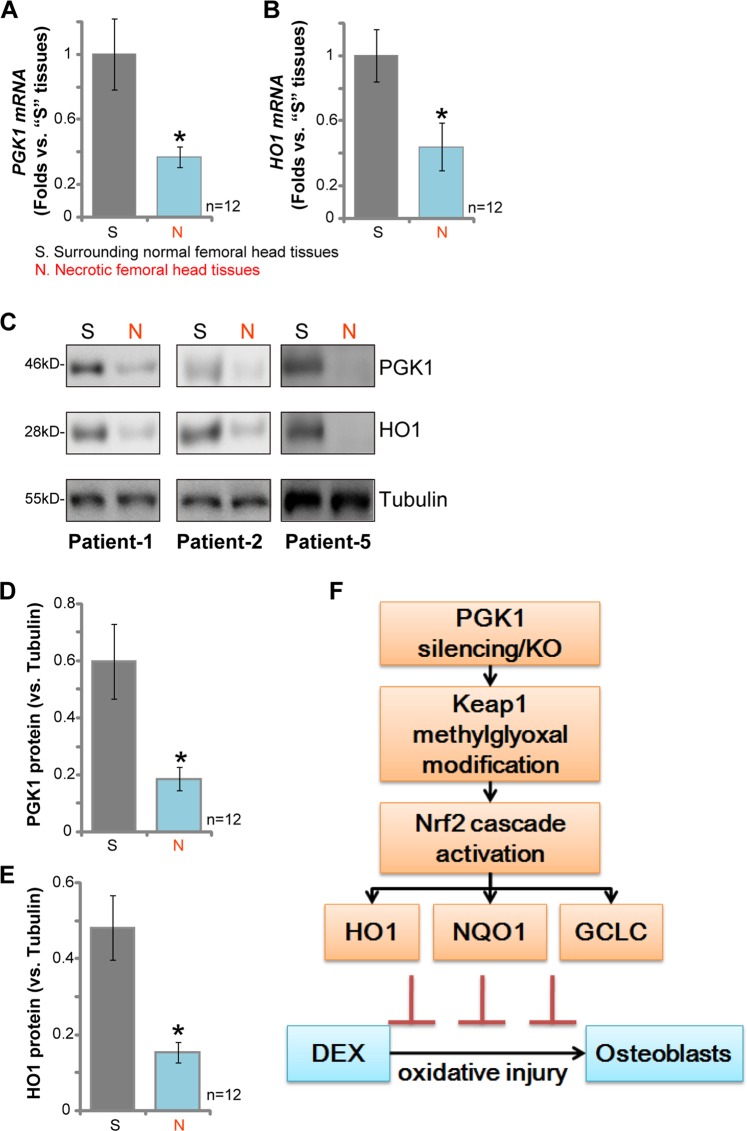
PGK1 downregulation in human necrotic femoral head tissues correlates with HO1 depletion. mRNA (**a**, **b**) and protein (**c**–**e**) expression of PGK1 and HO1 in necrotic femoral head tissues (“N”) and surrounding normal femoral head tissues (“S”) of 12 different DEX-taking patients was tested by qPCR and western blotting assays. **f** The proposed signaling carton of this study. Data were expressed as mean ± standard deviation (SD, *n* = 12). *p < 0.05 vs. “S” tissues. Experiments in this figure were repeated three times, and similar results obtained.

At last we tested PGK1 expression in the necrotic femoral head tissues of DEX-taking human patients. qPCR assay results, Fig. 6a, demonstrated that *PGK1 mRNA* levels were significantly downregulated in the necrotic femoral head tissues (“N”), when compared with its levels in the surrounding normal bone tissues (“S”). Importantly, *PGK1 mRNA* downregulated correlated with *HO1 mRNA* depletion in the necrotic femoral head tissues (Fig. 6b). Protein analyses, by western blotting, demonstrated that PGK1 and HO1 proteins were both downregulated in the necrotic femoral head tissues of representative patients (“Patient-1/-2/-5”, Fig. 6c). Statistical analyses integrating all 12 pairs of human tissues confirmed that PGK1 and HO1 protein downregulation in the necrotic femoral head tissues was significant (*p* < 0.05 vs. normal tissues) (Fig. 6d, e). Together, these results show that PGK1 downregulation in human necrotic femoral head tissues correlates with HO1 depletion.

## Discussion

Keap1 has reactive cysteine residues functioning as the electrophile sensor of reactive species^12,37^. Keap1’s covalent modification will induce Keap1-Nrf2 disassociation and Nrf2 accumulation due to decreased Nrf2 ubiquitination^12,37^. After nuclear translocation, the stabilized Nrf2 initiates the transcription and expression of antioxidant and other cytoprotective genes through binding to ARE loci^12,37^. Recent studies have discovered a direct link between glycolysis and the Keap1-Nrf2 cascade activation^19,38^. PGK1 is an essential enzyme required for the ATP-generating step in the glycolytic pathway^18^. PGK1 inhibition or depletion caused methylglyoxal accumulation, thereby modifying Keap1 to form a methylimidazole crosslink^19^. This will lead to Keap1 dimerization, Nrf2 accumulation and Nrf2 signaling activation^19^.

Here we demonstrated that PGK1 is functional expressed in OB-6 osteoblastic cells and primary human osteoblasts. PGK1 silencing, by targeted shRNA, resulted in Nrf2 cascade activation, leading to Nrf2 accumulation, nuclear translocation and expression of Nrf2-ARE-dependent genes, including *HO1*, *NQO1*, and *GCLC*. Furthermore, CRISPR/Cas9-induced PGK1 KO also led to significant Nrf2 cascade activation in OB-6 cells and primary osteoblasts. These results indicate that PGK1 depletion could be a novel and efficient strategy to activate Nrf2 signaling in human osteoblasts.

DEX usage is associated with increased risk of osteoporosis or even osteonecrosis in more than half of patients on long-term corticosteroid therapies^39^. Studies have shown that DEX will induce free radical toxicity and oxidative injury to osteoblastic cells/osteoblasts, causing significant cell apoptosis and necrosis^8,24,40^. Conversely, ROS scavenging will efficiently protect osteoblastic cells/osteoblasts from DEX^14,40,41^. Here we show that PGK1 shRNA in OB-6 cells and primary osteoblasts potently inhibited DEX-induced ROS production, cell apoptosis and programmed necrosis. Similarly, PGK1 KO also exerted osteoblast cytoprotection against DEX-induced oxidative injury. Thus, targeting PGK1 can protect human osteoblasts from DEX through inhibiting oxidative stress (see the proposed signaling pathway in Fig. 6f).

Significantly, we show that Keap1-Nrf2 signaling activation is required for osteoblast cytoprotection by PGK1 depletion. In OB-6 cells PGK1 KO-induced cytoprotection against DEX was almost completely reversed by Nrf2 shRNA. In addition, Keap1 silencing, by targeted shRNA, activated Nrf2 signaling and protected OB-6 cells from DEX, mimicking PGK1 depletion-induced actions. More importantly, Keap1 shRNA-induced Nrf2 activation and anti-DEX osteoblast cytoprotection were not further augmented with PGK1 silencing. These results clearly show that activation of Nrf2 cascade by PGK1 depletion protected osteoblastic cells/osteoblasts from DEX-induced oxidative injury. Although the detailed mechanisms may warrant further characterizations.

One important finding of this study is that *PGK1 mRNA* and protein expression is significantly downregulated in the necrotic femoral head tissues of DEX-taking patients. This could be one reason of Nrf2 signaling inhibition in necrotic femoral head tissues, as reported by other studies^6,32^. We further show that downregulation of PGK1 correlated with depletion of HO1, a key Nrf2 pathway gene^42^, in necrotic femoral head tissues of DEX-taking patients. The underlying mechanism of PGK1 downregulation shall need more studies.

## Conclusion

In summary we show that PGK1 depletion protects human osteoblasts from DEX via activation of Keap1-Nrf2 signaling cascade. Targeting PGK1-Nrf2 cascade could be a novel strategy to offer osteoblast cytoprotection against DEX-induced oxidative injury.
